# A Simple Yet Effective Preanalytical Strategy Enabling the Application of Aptamer-Conjugated Gold Nanoparticles for the Colorimetric Detection of Antibiotic Residues in Raw Milk

**DOI:** 10.3390/s22031281

**Published:** 2022-02-08

**Authors:** Víctor Díaz-García, Braulio Contreras-Trigo, Camila Rodríguez, Pablo Coelho, Patricio Oyarzún

**Affiliations:** Facultad de Ingeniería y Tecnología, Universidad San Sebastián, Sede Concepción, Concepción 4080871, Chile; bcontrerast@docente.uss.cl (B.C.-T.); rodriguezcamila@gmail.com (C.R.); pablo.coelho@uss.cl (P.C.)

**Keywords:** nanoaptasensor, preanalytical processing, clarification, antibiotics, gold nanoparticles, aptamer

## Abstract

The misuse of antibiotics in the cattle sector can lead to milk contamination, with concomitant effects on the dairy industry and human health. Biosensors can be applied in this field; however, the influence of the milk matrix on their activity has been poorly studied in light of the preanalytical process. Herein, aptamer-conjugated gold nanoparticles (nanoaptasensors) were investigated for the colorimetric detection in raw milk of four antibiotics used in cattle. The effect of milk components on the colorimetric response of the nanoaptasensors was analyzed by following the selective aggregation of the nanoparticles, using the absorption ratio A_520_/A_720_. A preanalytical strategy was developed to apply the nanoaptasensors to antibiotic-contaminated raw milk samples, which involves a clarification step with Carrez reagents followed by the removal of cations through dilution, chelation (EDTA) or precipitation (NaHCO_3_). The colorimetric signals were detected in spiked samples at concentrations of antibiotics as low as 0.25-fold the maximum residue limits (MRLs) for kanamycin (37.5 μg/L), oxytetracycline (25 μg/L), sulfadimethoxine (6.25 μg/L) and ampicillin (1 μg/L), according to European and Chilean legislation. Overall, we conclude that this methodology holds potential for the semiquantitative analysis of antibiotic residues in raw milk obtained directly from dairy farms.

## 1. Introduction

Bovine milk is one of the most important nutrient-rich food sources in the human diet [1], the global production of which reached 540,925 million tons in July 2021 [2]. The quality and safety of milk and dairy products are of paramount importance for the food industry and public health [3]. There is a rising concern surrounding the presence of antibiotic residues in raw milk, which are extensively used in dairy farms for the treatment and prevention of bacterial diseases affecting cattle as well as to improve animal performance [4]. Exposure to low levels of antibiotics in milk and milk derivatives is known to cause serious harmful effects in human health [5]. In addition, the usage of antibiotics in food-producing animals is a well-recognized factor in triggering the emergence of antimicrobial resistance, which contributes to select bacterial strains and antimicrobial resistance genes that can be further transferred to the human microbiome [6]. Bacterial multidrug resistance to antibiotics is today among the biggest threats to global health, accounting for over 700,000 deaths each year due to infections caused by antibiotic-resistant bacteria (ARB) [7]. ARB are expected to cause 10 million deaths per year by 2050 at an economic cost of USD 100 trillion [8]. Finally, antibiotic-contaminated raw milk may interfere with fermentation processes of dairy products by inhibiting the growth of lactic acid bacteria (starter cultures) [9].

The Codex Alimentarius Commission develops international food standards, including reference maximum residue limits (MRLs) for veterinary drugs. In the European Union (EU) the MRLs of antibiotic residues in milk are fixed by Commission Regulation N° 37/2010 [10], which are comparable to those set in Chilean legislation [11]. The Ministry of Health in Chile implements a national plan to control the presence of antibiotic residues in dairy products, while the Agriculture and Livestock Service (SAG, for its acronym in Spanish) is responsible for ensuring the sanitary inspection and control of veterinary pharmaceuticals in dairy cattle farms. Accordingly, raw milk contaminated with antibiotic residues at levels above the MRLs must be discarded to prevent them from entering the human food chain and their impacts on food safety and public health. Therefore, rapid screening kits (e.g., SNAPR beta-lactam tests) to monitor the presence of antibiotic residues in bovine raw milk have become a major requirement for farmers and the dairy industry. However, these tests are qualitative and unsuitable for the simultaneous detection of multiple groups of antibiotics. There is thus growing scientific and industrial interest in supporting the development of fast, sensitive and quantitative biosensing technologies to determine antibiotic residues in raw milk [12].

Nanotechnology is currently driving innovation in a pleyade of fields, including the sensing of environmental and food contaminants, industrial advanced materials and nanocatalysis applications in the chemical industry, as well as for water purification and the detection of explosives, among others [13,14,15,16,17]. Nanobiosensors are nanoscale sensors that include a biological recognition molecule (bioreceptor), which allow for improved analytical performances along with rapid and sensitive detection of analytes in the ppb (μg/L) concentration range [18,19]. A promising class of bioreceptors is DNA aptamers, which consist of short single-stranded oligonucleotides that provide high affinity and specificity for non-nucleotide molecules, including those with low molecular weight, toxic or nonimmunogenic [20]. The combination of AuNPs with aptamers in nanoaptasensors (NAS) is widely used to investigate the detection of a variety of analytes [21,22,23,24], with a growing number of reports addressing the detection of antibiotics in the field of food safety [25,26]. Surface plasmon resonance (SPR) is an optical property of AuNPs that allows the development of label-free NAS capable of analyzing multiple analytes in real time [27]. SPR causes a sharp and intense absorption band in the visible range, enabling colorimetric detection by following a red-to-purple–blue shift of the absorption spectrum during the aggregation of the nanoparticles. Optical NAS are a preferred sensing technique due to their non-invasive nature, high sensitivity, direct readout and easy coupling with other technologies, with recent developments in optical technology lowering the cost of the instrumentation [28,29]. However, most works address colorimetric detection exclusively from the point of view of the analytical behavior of the biosensor. Consequently, significant gaps remain in the literature regarding the preanalytical processes that are required to implement this technology in real-world applications with complex matrices.

Milk is a heterogeneous fluid composed of multi-dispersed phases of emulsion (fat–water), colloidal suspension (protein–water) and solution (salts–water), whose chemical complexity interferes with the analysis of antibiotic residues [30]. Previous reports shed light on the capacity of AuNP-based colorimetric aptasensors to detect a few of the antibiotic residues in commercial milk, including kanamycin [31], tetracycline [32], oxytetracycline [33] and streptomycin [34,35]. Preanalytical techniques described in these studies are often laborious and involve the use of chemical agents such as ethyl acetate, trichloroacetic acid or trifluoracetic acid. In addition, solvent extraction is especially challenging for nanobiosensor technology since pH and ionic environment severely influence their analytical behavior [36].

Herein, we investigated the effects of the main raw milk components on the activity of aptamer-conjugated AuNPs in the colorimetric detection of antibiotics belonging to four different groups used in cattle: kanamycin (aminoglycosides), oxytetracycline (tetracyclines), sulfadimethoxine (sulfonamides) and ampicillin (beta-lactams). A methodology was proposed to address preanalytical and analytical variables affecting the colorimetric detection of the four antibiotics in this matrix. The utility of this strategy was demonstrated according to the MRLs of veterinary drugs in food for human consumption accepted in EU and Chilean legislation.

## 2. Materials and Methods

### 2.1. Chemicals and Reagents

Kanamycin, oxytetracycline, sulfadimethoxine, ampicillin, tetrachloroauric acid solution (HAuCl_4_·3H_2_O), Carrez clarification reagent kit, Total Protein Kit, Micro Lowry reagent kit and Sephadex G-25 resin were purchased from Merk (Darmstadt, Germany). Ethyl acetate, lactose, ethylenediaminetetraacetic acid (EDTA), sodium citrate and all salt solutions were purchased from Winkler (Santiago, Chile). Aptamers were purchased from Integrated DNA Technologies, Inc. (Coralville, IA, USA).

### 2.2. Synthesis of Gold Nanoparticles (AuNPs)

The synthesis of AuNPs was carried out according to the standard citrate reduction method [24]. Briefly, 100 mL of 1 mM tetrachloroauric acid solution (HAuCl_4_·3H_2_O) was prepared with nanopure water (18 M Ω·cm). The solution was isovolumetrically heated to boiling point under stirring and refluxed with a three-neck round flask connected to the condenser. Then, 10 mL of 38.8 mM trisodium citrate solution at pH 11 was preheated to 60 °C and quickly added to the boiling solution of HAuCl_4_ under vigorous stirring [35]. After the color of the solution turned deep red the mixture was refluxed for an additional 30 min and cooled down to room temperature without stirring for 2 h. The resulting nanoparticle suspension was filtered with Millipore nylon filters (0.45 μm) and preserved in the dark at 4°C. The concentrations of AuNPs and NAS were calculated according to the Beer–Lambert law by measuring the absorbance at 520 nm (extinction coefficient of 2.01 × 10^8^ M^−1^ cm^−1^) [37,38].

### 2.3. AuNPs Characterization

The spectroscopic characterization of the synthesized AuNPs was carried out with an Epoch^TM^ microplate spectrophotometer (Biotek Instruments, Winooski, VT, USA). The size distribution and surface charge characterization (pZ) of the AuNPs were determined with a Zetasizer Nano-ZS90 dynamic light scattering (DLS) analyzer (Malvern Instruments, Westboroug, MA, USA). The characterization of the size and morphology of the nanoparticles was carried out by transmission electron microscopy (TEM) with a 4 Å resolution (TEM; JEOL-JEM 1200EX-II, Tokyo, Japan), using a Gatan CCD camera for image acquisition (model 782; Gatan, Inc., Pleasanton, CA, USA).

### 2.4. Synthesis of NAS and the Determination of Detection Parameters

AuNPs were functionalized with aptamers specific for the antibiotics listed in Table 1. Thiol-modified aptamers (C3-S-S-Aptamer) were reduced by incubation with dithiothreitol 0.1 M in a phosphate buffer of pH 8 for 3 h at 37 °C. Then, 3′-SH-aptamers were purified by gel filtration with Sephadex G-25 and incubated with the AuNPs in a phosphate buffer (10 mM, pH 7.4) for 2 days at room temperature and darkness [39]. AuNP–aptamer molar ratios of 1:20, 1:40 and 1:60 were investigated to determine the best parameters and conditions for the maximization of the colorimetric signal produced by the NAS in the detection of antibiotics at concentrations equal to their MRLs. The resulting NAS were activated by being heated at 80 °C for 10 min and then cooled down at room temperature for 10 min to induce linear conformation on the nanoparticle surface. This step is paramount to stabilize the nanoparticles and to allow the aptamers to interact with the antibiotics.

A typical assay in microplate wells consisted of 200 μL of antibiotic solution incubated with 100 μL of the activated NAS (4 nM) at 60 °C for 10 min, subsequently cooled down at room temperature. Then, 60 μL of NaCl 1 M was added into the solutions and incubated for 30 min to monitor the aggregation process. AuNP aggregation data were analyzed spectrophotometrically by measuring the shift of the plasmon resonance peak from 520 nm to 620 nm (A_520_/A_620_). The absorbance values were calibrated by subtracting the value in nanopure water (blank) and expressed as the difference in colorimetric signal between the control (without antibiotic) and the antibiotic solution, using concentration ranges around their MRLs (Equation (1)):

(1) $$\frac{A520}{A620}\mathrm{Control}-\mathrm{Treatment}=\mathrm{Signal}\frac{A520}{A620}\mathrm{NAS}\;\mathrm{without}\;\mathrm{antibiotics}\;-\;\mathrm{Signal}\frac{A520}{A620}\mathrm{NAS}\;\mathrm{with}\;\mathrm{antibiotics}\left(\mathrm{MRL}\right)$$

The aggregation of the NAS followed, during 60 min in a mixture of 100 μL of the activated NAS and 200 μL of nanopure water (without antibiotics). The absorbance was measured at 520 nm and 720 nm during 60 min (the absorption ratio A_520_/A_720_) upon the addition of the saline solution. Further experiments monitoring the SPR shift to 720 nm were also expressed according to Equation (1).

### 2.5. Preanalytical Processing of Raw Milk

Bulk tank raw milk samples (50 mL) were spiked with the antibiotics in final concentrations of 0.5×, 1×, 2× and 4× the corresponding MRLs and then homogenized for 30 min. The samples were clarified by using both Carrez reagents and ethyl acetate as follows:

- Carrez clarification: Five hundred milliliters of Carrez I reagent was added into 10 mL of raw milk without antibiotics (control) or with kanamycin, oxytetracycline, sulfadimethoxine or ampicillin, and vortexed for 1 min. Then, 500 mL of Carrez II reagent was added and vortexed for 1 min until the mixture was homogeneous, which was subsequently centrifuged at 1000× *g* for 5 min. The supernatant (milk whey) was recovered and immediately used for the detection of antibiotics with the NAS.
- Ethyl acetate clarification: Four milliliters of raw milk was centrifuged for 20 min at 1000 and 10 °C to separate the fat. Then, 2 mL of the supernatant was diluted with 2 mL of nanopure water and stirred for 10 min in a vortex. Seven milliliters of ethyl acetate was added, vortexed for 15 min and centrifuged for 15 min at 1500× *g* and 4 °C, which gave rise to a three-phase mixture. The bottom layer (milk whey) was recovered and centrifugated again before being stored at 4 °C.

### 2.6. Milk and Milk Whey Characterization

The proximate analysis of raw milk and whey (by Carrez method) was determined in accordance with the standard methods of the Association of Official Analytical Chemists (AOAC methods) [44]: fat (AOAC 945.16), ash (AOAC 920.181), crude fiber (AOAC 962.09), total protein (total nitrogen × 6.25) (AOAC 978.02), total carbohydrates (AOAC 929.09) and lactose (AOAC 982.14). The protein concentration was determined by the Lowry protein assay (Peterson’s modification) with protein precipitation, using the Total Protein Kit, Micro Lowry, in accordance with the manufacturer’s protocol. Optical density was measured at 650 nm in 96-well plates using and Epoch^TM^ microplate spectrophotometer. The protein removal efficiency was determined according to Equation (2):

(2) $$\mathrm{Protein}\;\mathrm{removal}\;\mathrm{efficiency}=\frac{C_{\mathrm{RM}}-{\;C}_{\mathrm{MW}\;}}{C_{\mathrm{RM}}}\;\text{×}\;100$$

where C_MW_ is the protein concentration of the milk whey and C_RM_ is the concentration of the raw milk.

### 2.7. Colorimetric Detection of Antibiotics in Clarificatecd Raw Milk

Ten milliliters of raw milk was spiked (contaminated) with kanamycin, oxytetracycline, sulfadimethoxine and ampicillin at final concentrations of 0.25×, 0.5×, 1×, 2× and 4× the MRL of each antibiotic and then incubated for 30 min at room temperature. The colorimetric detection of the antibiotics was thus investigated in solutions containing the main soluble components of bovine raw milk (lactose and ionic species) to determine their specific effect on the aggregation process of AuNPs (Table 2). Raw milk (with and without antibiotics) was clarified using the Carrez method, after which samples of the resulting whey were assessed with the NAS by following the aggregation according to the methodology described in Section 2.4.

### 2.8. Colorimetric Detection of Antibiotics in Cation-Removed Milk Whey

Raw milk samples were incubated with the antibiotics at concentrations of 0.25×, 0.5×, 1×, 2× and 4× their MRLs and subsequently subjected to Carrez clarification (see Section 2.5). Prior to performing the experiments of antibiotics detection the whey samples (1.5 mL) were divided into three isovolumetric fractions to remove cations by different methods: (i) by adding 80 μL of NaHCO_3_ 1 M into the solutions to a final concentration of 30 mM, which were subsequently incubated (10 min at 60 °C), cooled down at room temperature and centrifugated at 20,000× *g* for 10 min to recover the supernatant; (ii) by adding 80 μL of EDTA to a final concentration of 5 mM; and (iii) by diluting 1:1 with nanopure water to attenuate the effects of cations and lactose in the salt-induced aggregation of AuNPs. The absorbance values were normalized with respect to the absorbance ratio measured for the whey without antibiotics (control), according to the following equation:

(3) $$\mathrm{Fold}\;A520/A720=\frac{\mathrm{signal}\frac{A520}{A720}\mathrm{of}\;\mathrm{NAS}\;\mathrm{with}\;\mathrm{antibiotic}}{\mathrm{signal}\frac{A520}{A720}\mathrm{of}\;\mathrm{NAS}\;\mathrm{without}\;\mathrm{antibiotic}\;\left(\mathrm{control}\right)}$$

### 2.9. Statistical Analysis

Data shown are the average ± standard error of at least three independent experiments. Statistical significance was determined at a 95% confidence interval, using a nonparametric Mann–Whitney U test for the comparison of two groups.

## 3. Results and Discussions

### 3.1. NAS and Detection Principle

**Figure 1:**
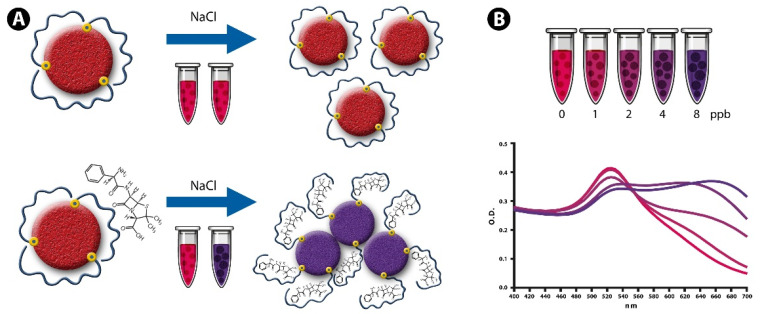
Schematic illustration showing the sensing principle of aptamer-conjugated AuNPs for the colorimetric detection of antibiotic residues present in raw milk. (**A**) Covalently conjugated aptamers in a random coiled linear structure inhibit salt-induced aggregation, while the conformational change induced by antibiotic interaction decreases surface protection and allows the aggregation of AuNPs upon the addition of NaCl. (**B**) Absorption spectra of AuNPs showing antibiotic-induced aggregation of the nanoparticles and the resulting shift in the plasmon resonance peak (from red to blue–purple).

The NAS developed herein are based on previously reported aptamers specific to four antibiotics used in cattle (kanamycin, oxytetracycline, sulfadimethoxine and ampicillin). Numerous works in the nanobiosensor field are based on aptamers bound to the nanoparticles through electrostatic interactions, which is a suitable approach for the detection of antibiotics in a saline buffer solution or highly clarified matrices. The recognition of antibiotics by adsorbed aptamers causes them to detach from the AuNPs with the concomitant loss of stability and subsequent aggregation [41]. However, this strategy becomes less applicable in complex matrices containing chemical species that can interfere with the analytical method. Accordingly, the experimental approach followed in this work involved the covalent conjugation of the ssDNA aptamers on the AuNPs’ surface (through thiol–gold interactions) to prevent their release from the nanoparticle surface. It is worth mentioning that this reaction has been well-characterized in numerous works, using different instrumental techniques such as X-ray photoelectron spectroscopy (XPS), X-ray diffraction (XRD) and atomic force microscopy (AFM). The resulting interaction between the thiol group and the gold surface is strong and stable, providing a robust mechanism to link aptamers onto AuNPs [45,46,47,48]. Importantly, the aptamers adopt a flexible random coil linear structure that allows their bases to interact with the nanoparticle surface through van der Waals forces [49]. Figure 1 provides a schematic description of the detection reaction, showing the aptamers coating the AuNPs’ surface and inhibiting salt-induced aggregation due to electrostatic repulsions among the nanoparticles. However, in the presence of the antibiotics the aptamers adopt a folded structure that leads to a decrease in surface protection and the subsequent aggregation of the AuNPs upon the addition of NaCl. The aggregation process is proportional to the antibiotic concentration and can be followed through the decrease in the absorption ratio (A_520_/A_620_).

### 3.2. Determination of the Detection Parameters of NAS

**Figure 2:**
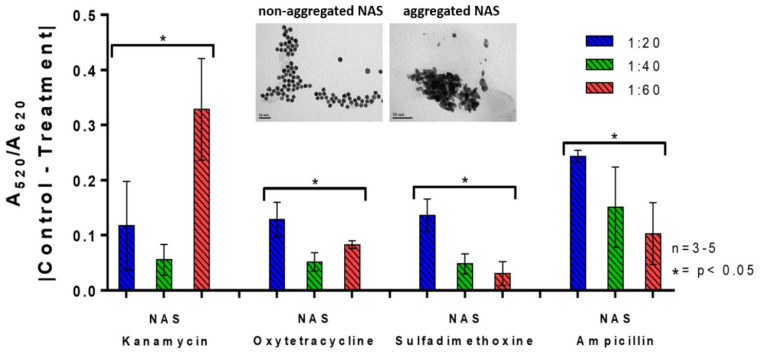
Colorimetric response of the NAS at molar ratios (AuNP:aptamer) of 1:20 (blue), 1:40 (green) and 1:60 (red) for the detection of antibiotics in water at their maximum concentration of residues permitted in milk (MRLs) for kanamycin, oxytetracycline, sulfadimethoxine and ampicillin. Treatments correspond to antibiotic-containing samples at the MRL concentrations and controls include nanopure water instead of antibiotics. The inset shows TEM images of dispersed NAS (before the detection of antibiotics) and aggregated NAS (after the detections of antibiotics). The absorbance readings were transformed according to Equation (1). Results were averaged from 3 to 5 independent experiments. Data were analyzed using a nonparametric Mann–Whitney U test. Asterisks denote statistically significant differences between the treatments and controls. * = *p* < 0.05.

The detection parameters of (i) AuNP:aptamer molar ratio; (ii) absorption ratio; and (iii) incubation time were evaluated in water to select conditions that maximized the colorimetric signal generated by the NAS. AuNP:aptamer molar ratios of 1:20, 1:40 and 1:60 were compared for each NAS in the presence of each antibiotic at concentrations equal to the corresponding MRL in the EU (Table 1). In the case of sulfadimethoxine the MRL considered was 25 μg/L (instead of 100 μg/L), which is the highest concentration of sulfonamides legally permitted in Chile for milk [11]. Differences in susceptibility to salt-induced aggregation at different molar ratios (AuNP functionalization) could be a consequence of the variable degrees of surface coverage in each NAS. Figure 2 shows the variation in the colorimetric signal produced by the NAS in detection of the four antibiotics upon the addition of NaCl. Dispersed and aggregated NAS resulting from the detection process are presented in Figure 2, with TEM images (inset) showing typical spherical particles with diameters of ~15 nm [24,27]. The highest colorimetric response was determined in the case of kanamycin at a molar ratio of 1:60, while for oxytetracycline, sulfadimethoxine and ampicillin the best molar ratio was 1:20. These parameters were selected for subsequent antibiotic detection assays.

We hypothesized that differences in the colorimetric response of NAS at different molar ratios are associated with the effect of this parameter on the degree of surface coverage of the AuNPs by the aptamers (i.e., reactive surface). Thus, nanoparticles partially coated on their surface would have fewer reactive sites available to interact with the antibiotics in comparison with mostly coated nanoparticles. By calculating the surface coverages for each mol of NAS and considering the aptamer length for NAS-Kan (21b), NAS-Oxy (76b), NAS-Sul (22b) and NAS-Amp (19b), the total number of moles of DNA bases that would be interacting with each NAS is 1260b (NAS-Kan), 1520b (NAS-Oxy), 440b (NAS-Sul) and 380b (NAS-Amp) (Table S1). Interestingly, both NAS-Amp and NAS-Sul share a low total number of bases available on their surfaces and a lower concentration of antibiotics in the assays (MRLs of 4 μg/L and 25 μg/L, respectively) in comparison with NAS-Oxy and NAS-Kan. By contrast, the latter groups of NAS have a higher number of bases coating the nanoparticle surface and a higher concentration of antibiotics (100 μg/L and 150 μg/L, respectively) in common. This analysis lends support to the relationship between reactive surface and the analyte concentration, given that NAS with a low proportion of aptamer coverage are more likely to become saturated at low concentrations and vice versa. Kim et al., for example, explored different molar ratios for the colorimetric detection of oxytetracycline in water, finding an optimal molar ratio of 1:50. However, this work was conducted with aptamers bound to AuNPs via electrostatic interactions, and the authors discussed the need to determine in each NAS the best molar ratio for optimal detection [35].

**Figure 3:**
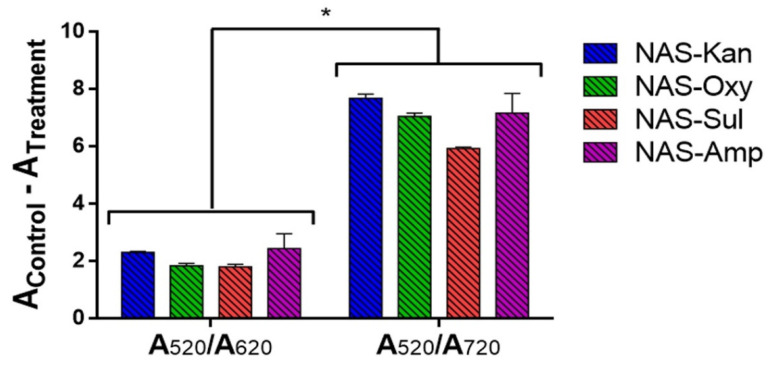
Comparison of the absorption ratios A_520_/A_620_ and A_520_/A_720_ from spectral variation associated with the aggregation of AuNPs as a colorimetric signal generated by the NAS in the detection of kanamycin (blue), oxytetracycline (green), sulfadimethoxine (red) and ampicillin (purple). Treatments correspond to antibiotic-containing samples at the MRL concentrations of each antibiotic, while controls include nanopure water instead of antibiotics. The absorbance readings were transformed according to Equation (1). Results were averaged from 3 to 5 independent experiments. Data were analyzed using a nonparametric Mann–Whitney U test. Asterisks denote statistically significant differences between the treatments and controls. * = *p* < 0.05.

The shift of the SPR peak (from 520 nm) associated with the aggregation of AuNPs spans a wide spectral region (until 800 nm), despite most of the works in the field of NAS following the shift between 520 nm and 620 or 650 nm [50]. Accordingly, our group previously developed an electro-opto-mechanic device for high-resolution AuNP spectral data in a wavelength range from 400 to 800 nm, which was proven to improve the analytical performance of NAS with the aid of machine learning tools [51]. With this in mind, AuNP aggregation data were analyzed spectrophotometrically between 400 and 750 nm, using two absorption ratios (A_520_/A_620_ and A_520_/A_720_) to select reading parameters that maximize the colorimetric signal associated with NAS detection and aggregation. As shown in Figure 3, the ratio A_520_/A_720_ outperformed A_520_/A_620_ in generating a color intensity three–four times greater in terms of the shift of the SPR peak. We proposed that the reason for this result is the greater difference between the absorbance values at each wavelength in the spectra before and after NaCl-induced AuNP aggregation. This phenomenon can be clearly seen in the Figure S1 of Supplementary Materials, where the differences in absorbance at 720 nm between the red (before aggregation) and blue (after aggregation) curves are greater than the difference at 620 nm. The relevant parameter is thus the difference between absorbances (not the absolute value); therefore, a greater difference at 720 nm is expected to decrease the detection limit by providing a stronger colorimetric signal. To the best of our knowledge this is the first study proposing the absorption ratio A_520_/A_720_ nm as a way to improve the sensitivity of the NAS.

**Figure 4:**
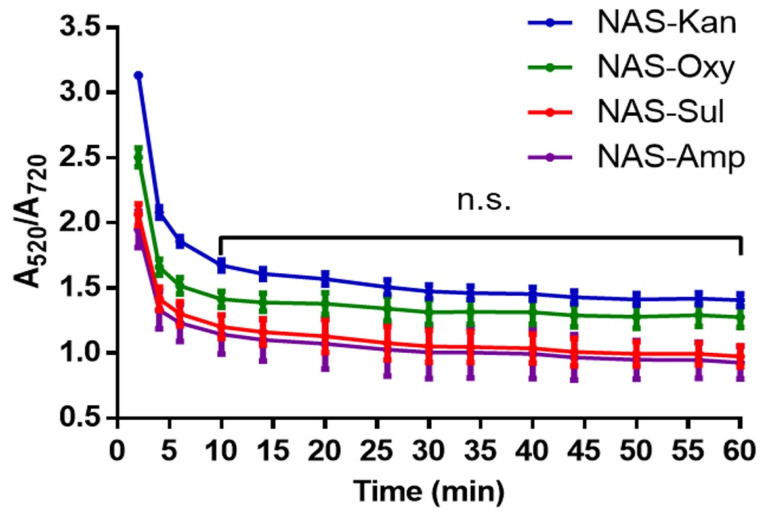
Kinetics of the aggregation of AuNPs in response to NAS detecting kanamycin (red), oxytetracycline (green), sulfadimethoxine (blue) and ampicillin (purple) upon the addition of NaCl. The results were averaged from 3 independent experiments for each NAS (*n* = 3). Non-significant differences are shown as n.s. Data were analyzed using a nonparametric Mann–Whitney U test.

Figure 4 presents the spectral variation across time resulting from the salt-induced aggregation of AuNPs for the four NAS. The curves exhibit an exponential decay, with a sharp decrease in the absorption ratio over the first 5 min followed by a slower exponential decline between 30 and 60 min. Based on these results the incubation time with NaCl was established as 30 min prior to proceeding with spectroscopic characterization.

**Figure 5:**
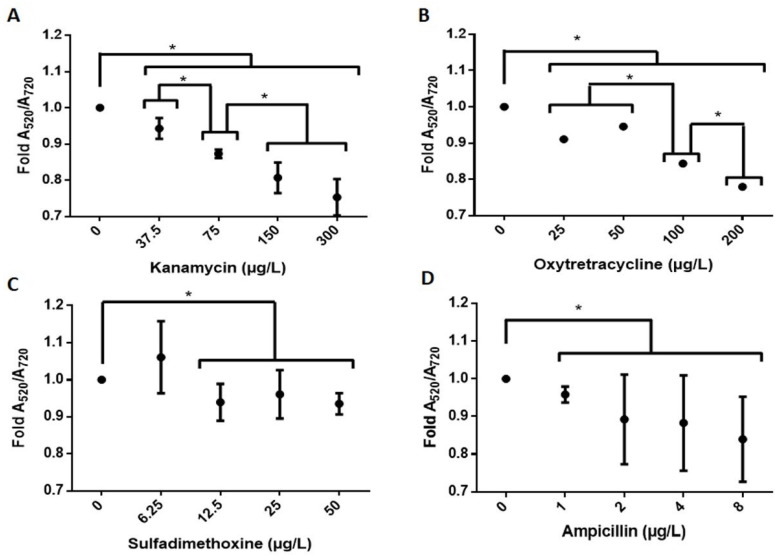
Colorimetric detection of the antibiotics in water for (**A**) kanamycin, (**B**) oxytetracycline, (**C**) sulfadimethoxine and (**D**) ampicillin, based on the A_520_/A_720_ absorption ratio. Results were averaged from three independent experiments (*n* = 3). Data were analyzed using a nonparametric Mann–Whitney U test. Asterisks denote statistically significant differences between the treatments and controls. * = *p* < 0.05.

The previously determined parameters were employed in antibiotic detection experiments. As shown in Figure 5, the NAS allowed for the discrimination of trace-level concentrations of the antibiotics kanamycin (0, 37.5, 75 and 150 μg/L), oxytetracycline (0, 50, 100 and 200 μg/L), sulfadimethoxine (above 12.5 μg/L) and ampicillin (above 1 μg/L).

These results suggest the need to include spectral information above 700 nm to better analyze the aggregation of AuNPs, in contrast with the most common approach of following the shift of the SPR band between 520 nm (red) and 620 or 650 nm (purple–blue) [37,41,42].

### 3.3. Raw Milk Clarification

The aggregation behavior of non-functionalized AuNPs in raw milk was initially determined in order to explore the activity of the NAS in this matrix. Figure S2 accounts for the absorption spectra of dispersed (~15 nm) and aggregated nanoparticles in the raw milk, which showed no spectral change in the plasmon resonance peak (at 520 nm) when incubating AuNP-containing raw milk with NaCl 0.1 M. The aggregation of AuNPs was followed through the A_520_/A_620_ ratio by increasing the NaCl concentration from 0.1 to 0.5 M. No significant differences between the control (without NaCl) and treated samples (with NaCl) were observed. The lack of aggregation of AuNPs in the milk suggests that the nanoparticles are strongly stabilized by electrostatic interactions with lactose or ion species, which interfere with the activity of NAS.

**Figure 6:**
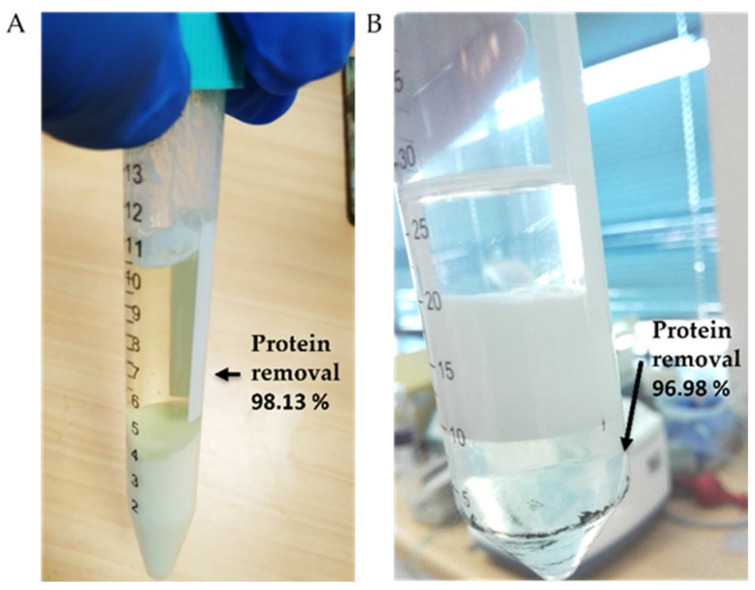
Phases and protein removal resulting from raw milk clarification using (**A**) the Carrez reagent and (**B**) ethyl acetate.

Two preanalytical treatments were employed for protein and fat removal from raw milk. The procedure based on the Carrez reagent produces two layers consisting of the supernatant (whey) and a precipitate of proteins and fat (Figure 6A). Instead, ethyl acetate forms three distinct layers corresponding to ethyl acetate (upper layer), a white layer of insoluble constituents (middle layer) and milk whey (bottom layer) (Figure 6B). The Carrez method outperformed in effectivity and simplicity the treatment with ethyl acetate to clarify raw milk, allowing around 98% of the protein content to be removed with the full elimination of fats (Table 3).

The Carrez method is often employed in the preanalytical processing of complex matrices. However, the absorption spectra of the four NAS in the resulting milk whey still showed no variations, accounting for a strong stabilization of the nanoparticles caused by ionic species in this solution (Figure S3). The NAS are prevented from generating a colorimetric response under these conditions, requiring further clarification steps.

### 3.4. Effect of Ion and Lactose on the Aggregation of AuNPs

In the milk serum sodium and potassium ions (Na^+^ and K^+^) form weak ion pairs with chloride, citrate and phosphate, mainly remaining as free ions while divalent cations (Ca^2+^ and Mg^2+^) are mostly complexed with citrate [52]. These cations are thus capable of interacting with negative charges of the citrate layer adsorbed onto AuNPs (citrate-capped AuNPs) and with phosphate groups from the DNA aptamers, thereby competing with electrostatic interactions that stabilize the nanoparticles and inducing their aggregation. This phenomenon occurs due to the displacement of the capping citrate layer and the consequent loss of the repulsive charges that counteract the van der Waal attractive forces between gold particles. Lactose, on the other hand, is the mayor carbohydrate component of milk and its interaction with AuNPs is also expected to influence aggregation. Given these complex interactions, synthetic milk whey (SMW) mimicking the ions and lactose composition of bovine raw milk was prepared with the aim of understanding the specific effects of these components on the activity of NAS.

**Figure 7:**
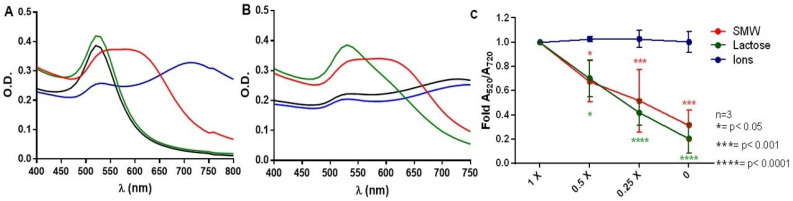
Spectroscopic characterization of the aggregation of AuNPs in synthetic whey (red line), lactose (green line), solution of ion species (blue line) and water (black line) during the incubation of AuNPs at (**A**) 10 min without the addition of NaCl addition and (**B**) 30 min after the addition of NaCl addition. (**C**) Effect of the solution dilution on the aggregation of AuNPs. The curves are based on the average of the results from three independent experiments (*n* = 3). Statistically significant differences compared with the controls and different treatments are indicated as * *p* < 0.05, *** *p* < 0.001 and **** *p* < 0.0001.

Figure 7 shows that the SMW (red line) and the ionic solution (blue line) induce the aggregation of AuNPs even before the addition of NaCl, according to the spectral shift of the plasmon resonance peak. The lactose solution (green line) prevented the salt-induced aggregation of AuNPs from taking place upon incubation both without NaCl (Figure 7A) and with NaCl (Figure 7B). This result suggests that lactose–AuNP interactions contribute to the stabilization of nanoparticles and would therefore inhibit salt-induced aggregation, a finding that is in agreement with previous works that show that this molecule is capable of interacting with metals cations such as iron [53] and calcium [54]. A recent study also reports the interaction of gold with the acetalized OH group of lactose [55]. Figure 7A,C point to the strong effect of milk ions on the aggregation of AuNPs as long as the aggregation of AuNPs caused by the ionic solution is similar to that induced by NaCl incubation, even decreasing the concentration of the ions four-fold. The activity of NAS becomes suppressed due to this effect (Figure S3).

The concentration of ions in the milk whey includes 132.423 mEq/L of cations (mono, di and trivalent) and 110.329 mEq/L of anions, where citrate and PO_4_^3−^ bind and chelate metallic cations in the solution (Table 2). Accordingly, ≈22.1 mEq/L of free cations are in theory involved in the aggregation of NAS. On the other hand, the aggregation of NAS induced by NaCl (monovalent cation) occurs at concentrations of 100 mEq/L of cations, while the aggregation of AuNPs starts at above 50 mEq/L (Figure S4). This analysis suggests that cations with a higher valence generate a stronger effect on the aggregation of AuNPs under equal conditions of normal concentration (Eq/L). Therefore, both cations and lactose need to be removed from the milk whey to make the aggregation of AuNPs possible and allow the NAS to be applied in this matrix.

### 3.5. Cation Removal and Antibiotic Detection

**Figure 8:**
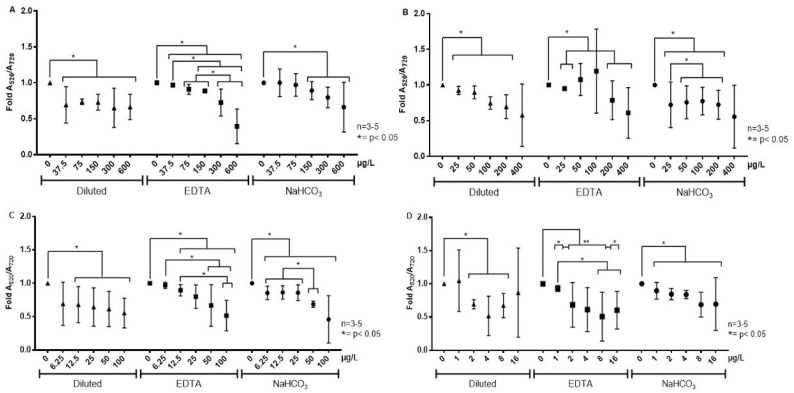
Colorimetric detection of antibiotics in cation-removed milk whey with NAS specific for (**A**) kanamycin, (**B**) oxytetracycline, (**C**) sulfadimethoxine and (**D**) ampicillin. The interferents were removed by dilution (▲), EDTA treatment (◼) and NaHCO_3_ treatment (●). The results were averaged from three independent experiments (*n* = 3). Statistically significant differences compared with the controls and different treatments are indicated. * *p* < 0.05 and ** *p* < 0.01.

Figure 8 shows that both the dilution and treatment of milk whey with chelating (EDTA) and precipitating (NaHCO_3_) agents allow the NAS to colorimetrically detect the corresponding antibiotics in concentrations as low as 0.25× their corresponding MRL. EDTA acts by sequestering divalent cations present in whey (Ca^2+^ and Mg^2+^), while NaHCO_3_ precipitates Ca^2+^ (as CaCO_3_) and Mg^2+^ (as Mg(OH)_2_). The colorimetrical signal is inversely correlated with the concentration of the different antibiotics (0–600 μg/L), where a decrease in the signal accounts for a larger presence of antibiotics in raw milk. Therefore, preanalytical steps must be considered as a part of the protocol to eliminate interference effects of cationic species, though the specific treatment with EDTA or NaHCO_3_ would depend on the antibiotic.

As summarized in Table 4, the methods employed to remove cations from the milk whey (dilution, chelation or precipitation) enabled the NAS to perform the sensitive detection of kanamycin (from 37.5 μg/L), oxytetracycline (from 25 μg/L), sulfadimethoxine (from 6.25 μg/L) and ampicillin (from 1 μg/L) in the clarified matrix. However, some of the treatments are associated with a higher degree of data dispersion (colorimetric signal) and these differences were further analyzed to select the best preanalytical methodology. In the case of kanamycin, the EDTA treatment allowed a highly sensitive detection to be achieved and qualitative discrimination between concentrations lower and higher than its MRL (150 μg/L). By contrast, the dilution and NaHCO_3_ treatments only allowed for the detection of the presence of antibiotics at the MRL concentration. For oxytetracycline both the dilution with water and NaHCO_3_ treatments enabled sensitive detection (from 25 to 200 μg/L), even though dilution was associated with less data variation and thus with a better detection performance. The NaHCO_3_ treatment was also suitable to perform the sensitive detection of sulfadimethoxine (from 6.25 μg/L) and to discriminate concentrations under the MRL and up to two-fold the MRL (50 μg/L). Instead, the dilution and EDTA treatments allowed for detection (from 12.5 μg/L) but without discrimination capability. Finally, the EDTA and NaHCO_3_ treatments were associated with the sensitive detection of ampicillin (from 1 μg/L). However, EDTA outperformed NaHCO_3_ in discriminating among different concentrations, despite it producing a rather high data dispersion. Therefore, the methodologies developed herein provide suitable conditions to discriminate at least 0.25 times the MRLs of each antibiotic, making it possible to implement the semiquantitative detection of them in samples of contaminated raw milk.

**Figure 9:**
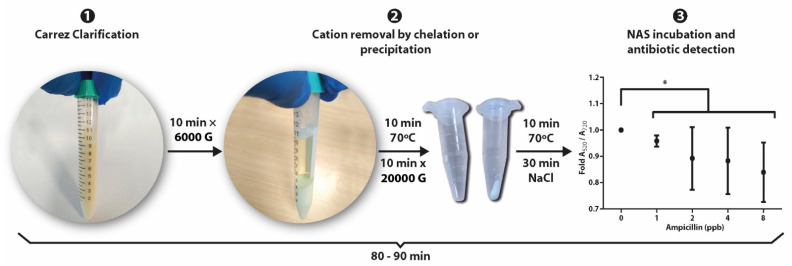
Preanalytical and analytical steps of the methodology developed to apply NAS technology for the detection in raw milk of kanamycin, oxytetracycline, sulfadimethoxine and ampicillin at MRL concentration levels.

In summary, the experimental strategy developed in this work involves three simple steps: (i) removal of fats and proteins with the Carrez reagents; (ii) elimination of ionic interferents by either dilution, chelation (EDTA) or precipitation (NaHCO_3_), depending on the target antibiotic; and (iii) incubation with the NAS to generate the colorimetric response (Figure 9).

Table 5 shows that the methodology developed reached low detection limits (nM range) and assay times (70 min) that are comparable with similar methods, but avoided the use of organic solvents and the requirement of complex pretreatment procedures. The proposed approach allowed for the gain of knowledge on the influence of the milk matrix on NAS activity, focusing on the preanalytical strategy required to implement the detection of antibiotic residues in raw milk directly obtained and assayed in a dairy farm. In addition, this is the first study addressing the effect of raw milk constituents on the colorimetric response of NAS in the process of detecting antibiotics belonging to four different groups (aminoglycoside, tetracycline, sulfonamide and beta-lactam).

In contrast to our approach, previous works report the determination of antibiotics on processed (commercial) milk, which is typically subjected to treatments involving the use of chemical agents such as ethyl acetate [43], trichloroacetic acid [31,33] or trifluoracetic acid [35], followed by centrifugation/resuspension. In some cases, final steps involve the adjustment of pH or the removal of solvents by nitrogen blow-down [57]. Milk dilution has also been reported elsewhere as a simple clarification procedure to deal with matrix complexity by using dilution factors ranging from 5-fold [35] to 50-fold [34]. However, in these works milk samples are typically spiked with antibiotics after dilution.

A realistic condition to apply NAS technology must consider the analyte already present in the sample of raw milk, because dilution could lower its concentration below the detection limit of the method. However, with the exception of Zhou et al. [31] the approach followed in the rest of the works consists of contaminating the milk after and not before the clarification step (Table 5). This is the reason why our work focuses on antibiotic-spiked raw milk samples, which were employed as the starting point from which to investigate a preanalytical strategy and analytical techniques necessary to advance NAS technology toward its application for real samples obtained in dairy farms. Table 5 highlights that this is the first study addressing this problem in raw milk, while other works have focused on milk powder [32], supermarket milk [35,43] or non-specified information [31,34,56]. Finally, a number of approaches have been proposed to generate and scale-up, at low costs, the production of AuNPs, as well as for the recovery of gold from laboratory wastes [58,59], which together contribute to the economic viability of this technology at an industrial level.

## 4. Conclusions

The contamination of raw milk with antibiotic residues is an issue of worldwide concern due to its impacts on the dairy industry and human health. The application of NAS for the detection of antibiotics in bovine raw milk involves preanalytical and analytical challenges related with the chemical complexity of this matrix. In this context, the bioconjugation of AuNPs with thiolated ssDNA aptamers provided suitable experimental conditions with which to apply NAS for the colorimetric detection of antibiotics in raw milk. For the first time a methodology was proposed to enable the highly sensitive colorimetric detection of antibiotics belonging to four different groups, namely kanamycin (aminoglycosides), oxytetracycline (tetracyclines), sulfadimethoxine (sulfonamides) and ampicillin (beta-lactams), using a straightforward preanalytical process. The method developed herein consists of a clarification treatment with Carrez reagents, followed by the removal of cations from milk whey through dilution, chelation (EDTA) or precipitation (NaHCO_3_). In all cases the methodology allowed for the semiquantitative detection of the colorimetric signals generated by the NAS at concentrations as low as 0.25-fold the MRL of the antibiotics. However, the specific treatment to address ionic interference (dilution/chelation/precipitation) depends on the particular antibiotic to be determined in the raw milk. Furthermore, the results proved that analyzing the spectral shift at 720 nm (A_520_/A_720_) improved the analytical performance of the NAS in comparison with the typical absorption ratio (A_520_/A_620_). Overall, this methodology combines simplicity and sensitivity for the four antibiotics, holding the potential to be applied for semiquantitative analyses of antibiotic residues in raw milk obtained directly from dairy farms.

## Figures and Tables

**Table 1:** Antibiotics used in the experiments, including their maximum acceptable levels in raw milk (MRLs) according to EU and Chilean legislation, and aptamers used in the NAS to recognize each antibiotic.

Antibiotic	Maximum Residual Limit (MRL)	Aptamer Sequence (ssDNA)	Aptamer Ref.
Kanamycin	150 μg/L *	5′-TGGGGGTTGAGGCTAAGCCGA-3′ (21b)	[40]
Oxytetracycline	100 μg/L *	5′-CGTACGGAATTCGCTAGCGGGCGGGGGTGCTGGGGGAATGGAGTGCTGCGTGCTGCGGGGATCCGAGCTCCACGTG-3′ (76b)	[41]
Sulfadimethoxine	25 μg/L ^†^ 100 μg/L *	5°′-GAGGGCAACGAGTGTTTATAGA-3′ (22b)	[42]
Ampicillin	4 μg/L *^,†^	5′-GCGGGCGGTTGTATAGCGG-3′ (19b)	[43]

* EU legislation [10]. ^†^ Chilean legislation [11].

**Table 2:** Solutions employed to study the effect of soluble milk constituents on the aggregation of the NAS.

Element	Concentration	Synthetic Milk Whey	Lactose Solution	Ionic Solution
Lactose	5% ^w^/_v_	X	X	
Ca^2+^	30.120 mM (60.232 mEq/L)	X		X
Mg^2+^	4.750 mM (9.506 mEq/L)	X		X
Fe^3+^	0.011 mM (0.032 mEq/L)	X		X
PO_4_^3−^	30.582 mM (91.745 mEq/L)	X		X
Na^+^	23.587 mM (23.587 mEq/L)	X		X
K^+^	38,930 mM (38.930 mEq/L)	X		X
Zn^2+^	0.066 mM (0.131 mEq/L)	X		X
Citrate^3−^	9.292 mM (18.584 mEq/L)	X		X
Cu^2+^	0.002 mM (0.005 mEq/L)	X		X

The symbol (X) indicates the presence of each particular milk component in the solutions.

**Table 3:** Proximate analysis of raw milk and whey obtained with the Carrez method.

Components	Raw Milk	Milk Whey
Lipids	5.3%	0.0%
Proteins	3.2%	0.2%
Raw fiber	0.4%	0.6%
Total carbohydrates	4.7%	4.5%
Lactose	5.0%	5.3%
Ashes	0.7%	1.2%
pH	5.94	6.05

**Table 4:** Selected methods for the removal of cationic interferents, which provide the minimum detection limit of antibiotic for each NAS.

Nanoaptasensor	Best Method for Interference Elimination	Detection Limit
Kanamycin	EDTA	37.50 μg/L (0.25 MRL)
Oxytetracycline	Dilution	25.00 μg/L (0.25 MRL)
Sulfadimethoxine	NaHCO_3_	6.25 μg/L (0.25 MRL)
Ampicillin	EDTA	1.00 μg/L (0.25 MRL)

**Table 5:** Colorimetric AuNP-based aptasensors for the determination of antibiotics in milk.

Milk Sample	Antibiotic Residue	Preanalytical Time	Assay Time	LOD	Range	Pretreatment/Antibiotic Addition	Ref.
Supermarket milk	Ampicillin (AMP)	1.5 h	80 min	10 ng/mL (28.6 nM)	1–100 ng/mL (2.86–286 nM)	Ethyl acetate addition/centrifugation at 5000 rpm to obtain supernatant/nitrogen blow-down at 40 °C/pellet resuspension in water (total dilution: 2-fold)/**AMP addition (after pretreatment)**.	[43]
N.S.	Tetracycline (TET)	N.S.	25 min	45.8 nM	10–400 nM	Milk dilution with water (1:5)/acetic acid addition/centrifugation (total dilution: 5-fold)/**TET addition** (protocol details unspecified).	[56]
Milk powder	Tetracycline (TET)	N.S.	60 min	122 nM	10–500 nM	Acetic acid addition/centrifugation/**TET addition** (protocol details unspecified).	[32]
N.S.	Kanamycin (KAN)	35 min	100 min	1 nM	1–8 nM 100–500 nM	**KAN addition (before pretreatment)**/acetic acid addition/incubation at 45 °C/centrifugation at 10,000 rpm/filtration 0.22 μm/pH adjustment (total dilution unspecified).	[31]
N.S.	Streptomycin (STR)	N.A.	70 min	73.1 nM	30–1030 nM	Milk dilution with water (total dilution: 50-fold)/**STR addition (after pretreatment).**	[34]
Supermarket milk	Streptomycin (STR)	30 min	60 min	86 nM	100–500 nM	Milk dilution with water (total dilution: 5-fold)/**STR addition (after dilution)**/EDTA and trifluoracetic acid addition/centrifugation at 6000 rpm/supernatant collection.	[35]
Raw milk from a dairy farm	Ampicillin Oxytetracycline Sulfadimethoxine Kanamycin	40 min	30 min	1 μg/L (2.9 nM) 25 μg/L (54.3 nM) 6.25 μg/L (20.9 nM) 37.5 μg/L (77.4 nM)	1–16 μg/L (2.9–46.4 nM) 25–200 μg/L (54.3–434.4 nM) 6.25–100 μg/L (20.9–334.4 nM) 37.5–600 μg/L (77.4–1238.4 nM)	**Antibiotic addition (before pretreatment)**/Carrez reagents/centrifugation at 1000 rpm/dilution, NaHCO_3_ or EDTA treatments/centrifugation at 1000 rpm/supernatant collection (total dilution: 1.16-fold).	This study

N.S. = Details not specified by the authors. N.A. = Not applicable.

## Data Availability

The data presented in this study are available on request from the corresponding author. The data are not publicly available due to privacy restrictions.

## Supplementary Materials

The following are available online at https://www.mdpi.com/article/10.3390/s22031281/s1, Figure S1: Spectral variation associated with the aggregation of AuNPs of the NAS, Figure S2: Aggregation of AuNPs in raw milk, Figure S3: Detection of antibiotics in milk whey using the NAS, Figure S4: Aggregation of AuNPs versus NAS in water, Table S1: Molar ratio between AuNPs-aptamers and AuNPs-aptamers bases.
