# Supplementary material for: A Simple Yet Effective Preanalytical Strategy Enabling the Application of Aptamer-Conjugated Gold Nanoparticles for the Colorimetric Detection of Antibiotic Residues in Raw Milk
Source: Sensors (Basel). 2022 Feb 8;22(3):1281. doi: 10.3390/s22031281 (PMC8837955; doi:10.3390/s22031281)
Supplement: Supplementary file 1 [file sensors-22-01281-s001.zip › sensors-1518354-supplementary.pdf]

Supplementary Material

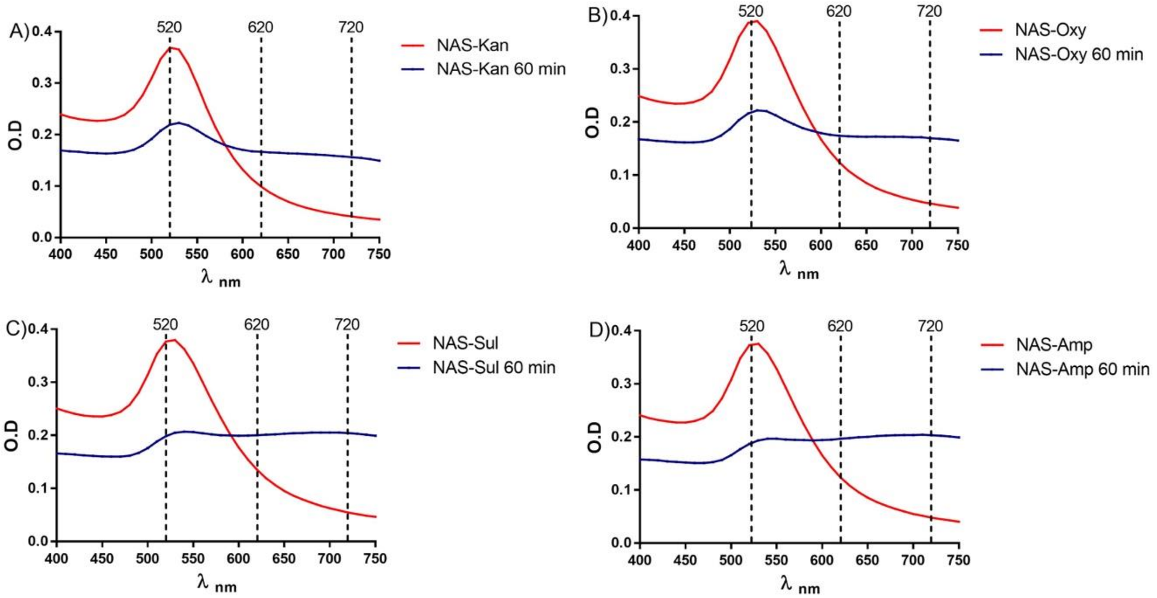

Figure S1. Spectral variation associated with AuNPs aggregation of nanoaptasensors detecting kanamycin (A), oxytetracycline (B), sulfadimetoxin (C) and ampicillin (D) upon NaCl addition. The results were averaged from independent experiments for each of nanoaptasensor (n=3).

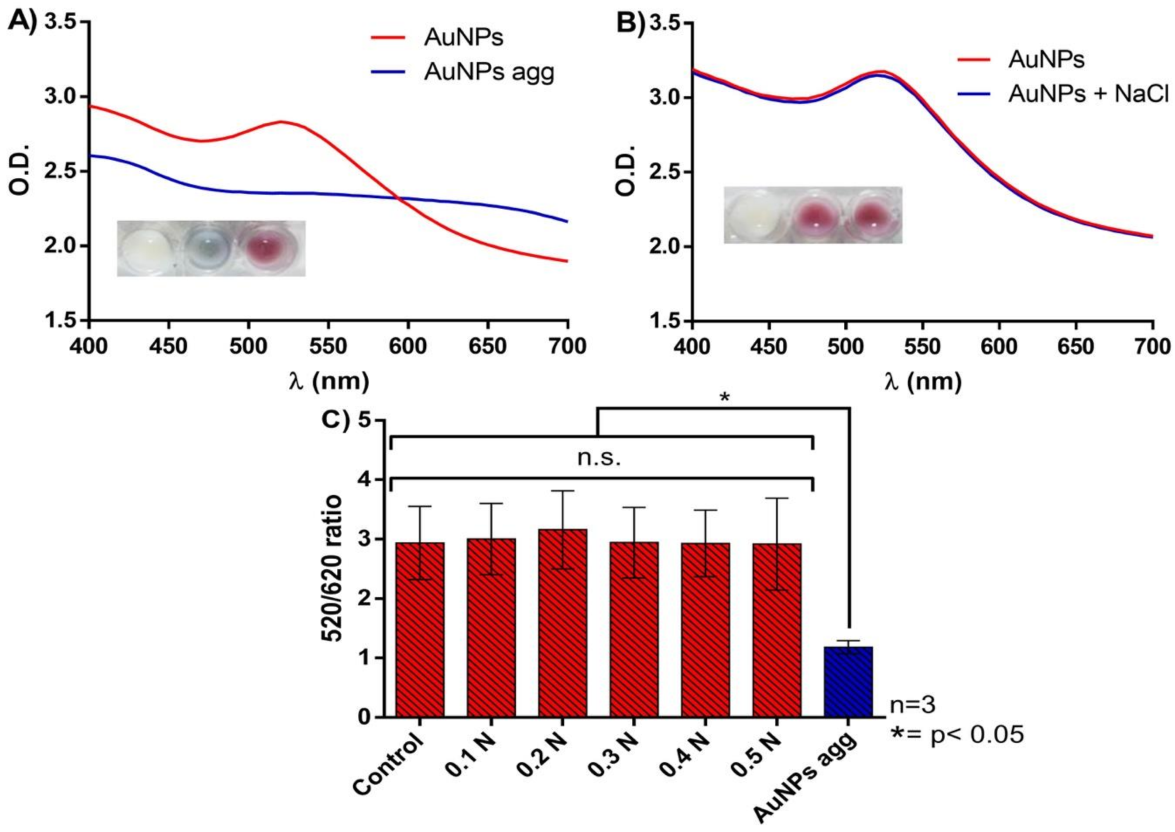

Figure S2. AuNPs aggregation in raw milk. A) Absorption spectrum resulting from addition to raw milk of dispersed AuNPs (red) and aggregated AuNPs (blue). The inset image shows microwells containing raw milk without AuNPs (blank), with aggregated AuNPs (blue) and with dispersed AuNPs (red); B) Spectrum of AuNPs in raw milk before (red) and after (blue) addition of NaCl 1 M to induce nanoparticle aggregation (0.1 N NaCl final concentration). Inset image shows microwells containing raw milk (blank), raw milk containing AuNPs with (blue) and without (red) addition of NaCl 1 N; C) Spectral changes upon aggregation of AuNPs in raw milk (A_{520}/A_{620}) at NaCl concentrations ranging from 0.1 to 0.5 N. Results were averaged from three independent experiments (n=3). Data were analyzed using the nonparametric Mann–Whitney test. Statistically significant differences compared with the controls and different treatments are indicated. * = $p< 0.05$.

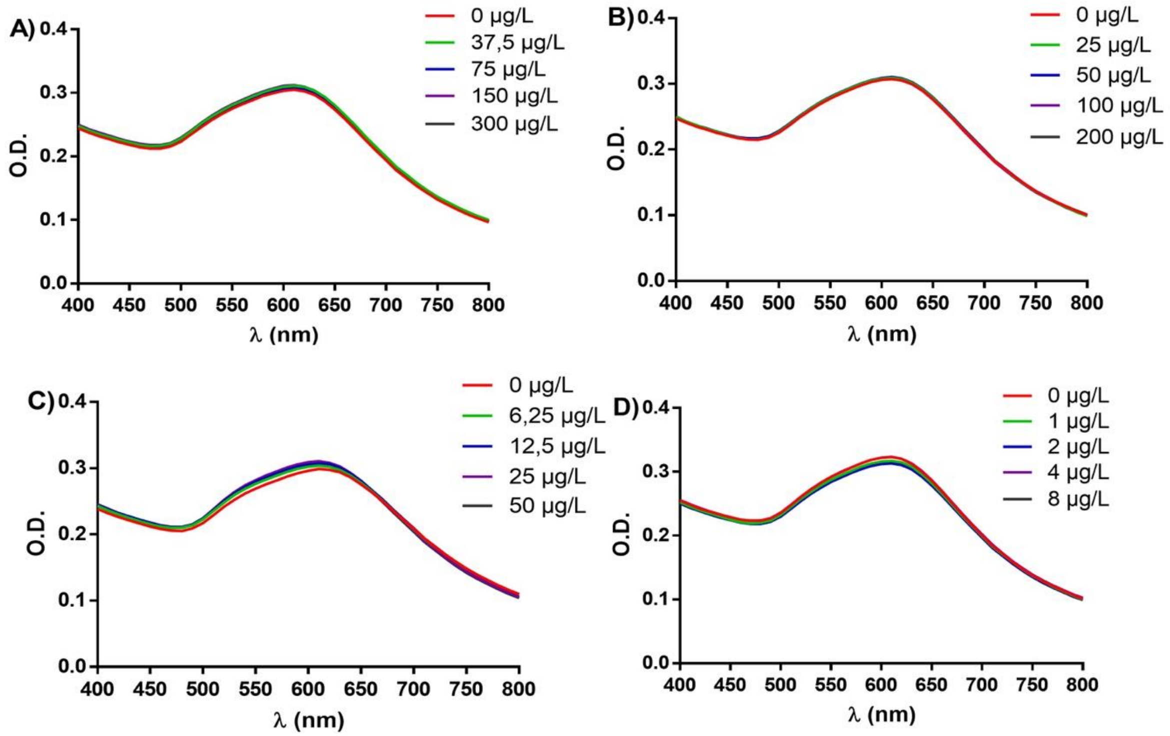

Figure S3. Absorption spectra of the nanoaptasensors in raw milk whey in colorimetric detection of the antibiotics at different concentration ranges: (A) NAS-Kan for kanamycin (0-300 ppb); (B) NAS-Oxy for oxytetracycline (0-200 ppb); (C) NAS-Sul for sulfadimethoxine (0-50 ppb) and (D) NAS-Amp for ampicillin (0-8 ppb). Detection was performed as previously described in subsection 2.4 (Materials and Methods). The curves correspond to the average of three independent experiments (n = 3).

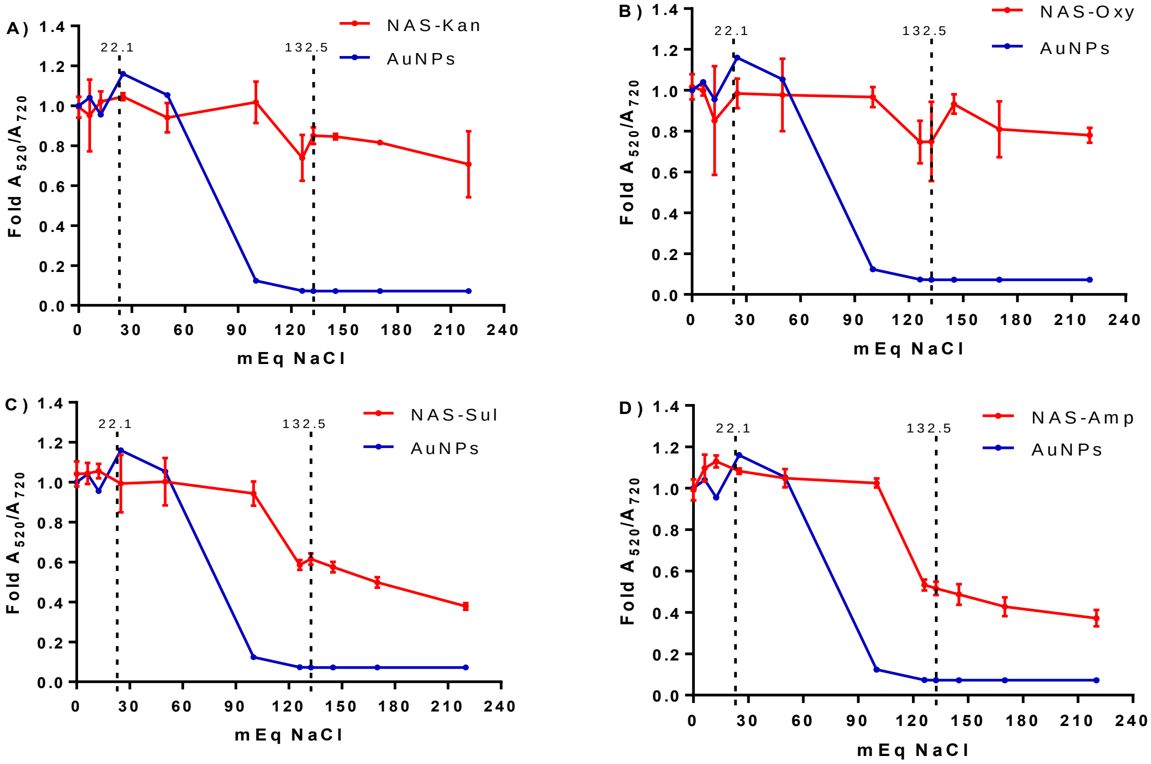

Figure S4. Aggregation of AuNPs versus NAS in water. Different NAS and AuNPs were incubated with 0, 6.25, 12.5, 25, 50, 100 126.25, 132.5 145, 170 and 220 mEq of NaCl by 30 min. Aggregation was followed in terms of the variation of fold at A_{520}/A_{720} ratio of AuNPs with NaCl compared with treatments without NaCl. Segmented line indicates the concentration of total cations (132.5 mEq) and free cations (22,1 mEq/L) on milk whey.

Table S1: Molar ratio between AuNPs-aptamers and AuNPs-aptamers bases

NAS	Molar Ratio AuNPs : aptamers	Aptamer size	Molar Ratio AuNPs : bases
Kan	1:60	21 bases	1: 1260
Oxy	1:20	76 bases	1: 1520
Sul	1:20	22 bases	1: 440
Amp	1:20	19 bases	1: 380
